# Microsporidia (*Encephalitozoon cuniculi*) in Patients with Degenerative Hip and Knee Disease, Czech Republic

**DOI:** 10.3201/eid3003.231263

**Published:** 2024-03

**Authors:** Bohumil Sak, Petra Gottliebová, Elka Nyčová, Nikola Holubová, Jana Fenclová, Marta Kicia, Żaneta Zajączkowska, Martin Kváč

**Affiliations:** Biology Centre of the Czech Academy of Sciences, České Budějovice, Czech Republic (B. Sak, N. Holubová, J. Fenclová, M. Kváč);; Bulovka Hospital, Prague, Czech Republic (P. Gottliebová, E. Nyčová);; University of South Bohemia, České Budějovice (J. Fenclová, M. Kváč);; Wroclaw Medical University, Wroclaw, Poland (M. Kicia, Ż. Zajączkowska)

**Keywords:** Encephalitozoon cuniculi, microsporidia, hip, knee, PCR, qPCR, arthroplasty, prosthetic joint infection, implant loosening, parasites, zoonosis, Czech Republic

## Abstract

Total joint arthroplasty is a commonly used surgical procedure in orthopedics. Revision surgeries are required in >10% of patients mainly because of prosthetic joint infection caused by bacteria or aseptic implant loosening caused by chronic inflammation. *Encephalitozoon cuniculi* is a microsporidium, an obligate intracellular parasite, capable of exploiting migrating proinflammatory immune cells for dissemination within the host. We used molecular detection methods to evaluate the incidence of *E*. *cuniculi* among patients who had total hip or knee arthroplasty revision. Out of 49 patients, *E. cuniculi* genotypes I, II, or III were confirmed in joint samples from 3 men and 2 women who had implant loosening. Understanding the risks associated with the presence of microsporidia in periprosthetic joint infections is essential for proper management of arthroplasty. Furthermore, *E. cuniculi* should be considered a potential contributing cause of joint inflammation and arthrosis.

Microsporidia are a group of obligate intracellular parasites comprising ≈1,300 species within >200 genera (*1*). Microsporidia are considered to be closely related to fungi (*2*,*3*) and infect a broad range of invertebrates and vertebrates, from protists to humans (*4*). Because of improved detection methods and greater awareness, microsporidia have been detected in a broad range of human populations, including children, travelers, elderly persons, and organ transplant recipients (*5*). Persons with high exposure to animals and contaminated soil and water are considered at risk for microsporidiosis (*6*). Of the several species of microsporidia that infect humans, *Encephalitozoon cuniculi* is the most common (*7*). Four genotypes of *E. cuniculi* have been identified on the basis of variable repeats in the rRNA internal transcribed spacer; however, human infections are mostly associated with genotypes I and II (*8*).

The digestive tract is an entrance point for microsporidia and subsequent spreading of infection occurs in all parts of the intestine. Within weeks, infection spreads to other tissues and organs, most commonly the kidney, liver, spleen, lung, and brain, depending on the species-specific interaction with the host (*8*). However, the unique mechanism of host cell invasion involving a highly specialized structure, the 10–50 μm long polar filament, enables only limited spread over short distances within the host. Therefore, the dissemination rate suggests the possible engagement of macrophages or other immune cells involved in inflammatory responses, which can serve as vehicles transporting microsporidia to foci outside of the intestine (*9*,*10*). Microsporidia are often overlooked in clinical samples because of problematic diagnoses, increasing the likelihood of hidden infections that can cause extensive tissue damage and various nonspecific pathologies and that often go without effective treatment (*11*).

Total joint arthroplasty is one of the most common surgical procedures in orthopedics to replace joints in patients with degenerative diseases (*12*). Revision surgeries are required in >10% of those patients because of implant failure caused mainly by prosthetic joint infection and aseptic implant loosening from inflammation (*13*,*14*). Whereas prosthetic joint infection is caused by bacterial infection (e.g., *Staphylococcus aureus*, *Streptococcus* spp., and *Enterococcus faecalis*) and pathologic growth around the prosthetic joint (*15*), aseptic implant loosening results from chronic inflammation caused by activation of resident immune cells in contact with implant wear debris or allergic reactions to metal ions derived from implant materials (*16*). However, the classification of aseptic implant loosening might be misleading because other pathogens are often overlooked, and the condition is potentially mislabeled as aseptic (*17*).

*E. cuniculi* is considered a cause of osteolysis in hip periprosthetic tissue (*17*), and connections between proinflammatory immune responses and concentration of *E. cuniculi* in inflammatory foci have been reported (*9*,*10*). Understanding the risks for microsporidiosis within periprosthetic joints is essential for proper arthroplasty management. We evaluated the incidence of generally neglected microsporidia among patients who had total hip or knee arthroplasty revision. 

## Methods

### Patients

We investigated samples obtained from immunocompetent patients who were hospitalized or who visited the orthopedic clinic at Bulovka Hospital (Prague, Czech Republic) during May 2020–September 2021. The first group of patients had undergone a hip puncture/total hip revision arthroplasty, and the second group had undergone a knee puncture/knee revision arthroplasty (in 1 case, the patient only underwent a knee arthroscopy). We assigned patients to 3 diagnostic groups according to microbiologic cultures and criteria of the Infectious Diseases Society of America or the Musculoskeletal Infection Society for periprosthetic joint infection according to the judgement of the treating physician: periprosthetic joint infection, aseptic implant loosening (aseptic loosening was diagnosed when signs of implant loosening were present, but infection was not the cause), and other diagnosis (patients who did not fit into the first 2 groups) (*18*,*19*).

### Sample Collection

Fragments of periprosthetic hip and knee tissues and joint fluids were collected intraoperatively; joint aspirates were collected during knee or hip punctures. Samples for microbiologic culture (i.e., samples of joint tissues, joint fluids, and surgical swabs from the endoprosthesis, tissues, or joints) were gathered intraoperatively. The number of samples collected was at the discretion of the orthopedic surgeon. Surgical swabs or other samples with insufficient volumes were excluded from the study. All samples were collected under sterile conditions. Each sample was placed in a separate sterile container and delivered at room temperature (20°–25°C) to the Department of Clinical Microbiology at Bulovka Hospital. Samples collected outside of laboratory working hours were maintained at room temperature (20°–25°C) overnight and then processed.

Samples were processed in a laminar flow cabinet for microbiologic culture; aliquots were stored without preservatives at −20°C for further molecular investigation and sent to the Biology Centre of the Czech Academy of Sciences for microsporidia screening. For *Neisseria* identification, we inoculated samples onto GO blood agar (LabMediaServis s.r.o., https://www.labmediaservis.cz) and 5% sheep blood agar, and incubated at 37°C in 5% CO_2_ for 24 h for GO blood agar and 48 h for 5% sheep blood agar. We cultured samples on Endo agar, in liver broth, and on sheep blood agar (containing 10% NaCl) at 37°C in an aerobic atmosphere for 24 h and 48 h (blood agar). After 24 h, we subcultured the liver broth on 5% sheep blood agar in a 5% CO_2_ atmosphere and Endo agar in an aerobic atmosphere for another 24 h. We examined cultures for bacterial growth after 24 h and 48 h (GO blood agar). If no growth occurred, we incubated the 5% sheep blood agar and GO blood agar cultures for 7 d. For anaerobic cultures, we inoculated patient samples onto Schaedler agar and in thioglycolate broth and cultured in an anaerobic atmosphere for 48 h and a total of 7 d. Depending on the microbiologist’s decision, we subcultured the thioglycolate broth cultures onto Schaedler agar. We identified all bacteria by using standard laboratory procedures, including biochemical testing, by using the BD Phoenix system (Becton Dickinson, https://www.bd.com), and, in the case of *Salmonella* Enteritidis, by serotyping. We performed antimicrobial drug susceptibility testing by using European Committee on Antimicrobial Susceptibility Testing methodology (https://www.eucast.org). We prepared fungal cultures on Sabouraud agar only when requested by the orthopedic surgeon; those plates were incubated aerobically at 37°C for 48 h, examined, and then cultured for a total of 7 d.

### DNA Isolation

We used aliquots of tissue and primary materials from joint aspirates and fluids from each patient for DNA isolation. We homogenized a total of 200 mg of tissue or aspirate sediment by using bead disruption on a FastPrep-24 instrument (MP Biomedicals, https://www.mpbio.com) at a speed of 5.5 m/s for 1 min. We extracted total DNA by using the DNeasy Blood and Tissue Kit (QIAGEN, https://www.qiagen.com) according to the manufacturer’s instructions. We included an extraction negative control to each DNA extraction series to ensure the absence of contamination in reagents, consumables, and the environment. We stored extracted DNA at −20°C until PCR amplification. We isolated control DNA from purified *E. intestinalis* spores by using the same methods.

### Molecular Examination

We amplified a partial sequence of the 16S rRNA gene that included the entire internal transcribed spacer by using nested PCR protocols with microsporidia-specific primers (*9*). We used DNA obtained from *E. intestinalis* spores as a positive PCR control and ultrapure water (without template) as a negative control in each PCR run. We evaluated the PCR products by gel electrophoresis.

We processed DNA from microsporidia PCR-positive samples by using a real-time quantitative PCR protocol that amplified a 268-bp region of the *E. cuniculi* 16S rRNA gene (*9*). We used negative controls comprising unspiked specimens and diluent blanks for each PCR. We determined positive results according to mathematical algorithms included with the LightCycler System (Roche, https://www.roche.com); results were positive when the cycle threshold was <43. We calculated the total number of spores in 1 g of sample according to a standard curve derived from spore DNA that was serially diluted in water; dilutions ranged from 1 to 1 × 10^8^ (R^2^ = 0.9903).

### Phylogenetic Analyses

We purified PCR amplicons by using the QIAquick Gel Extraction Kit (QIAGEN), and sequencing was performed in both directions at SeqMe (https://www.seqme.eu). Amplification and sequencing of each positive sample was repeated 3 times. 

We manually edited the nucleotide sequences by using ChromasPro 2.1.4 (Technelysium, https://www.technelysium.com.au) and aligned the sequences with references from GenBank by using MAFFT version 7 (http://mafft.cbrc.jp). We performed phylogenetic analysis by using the maximum-likelihood method and evolutionary models selected by MEGA X software (MEGA, https://www.megasoftware.net). We inferred the evolutionary history for partial sequences of the 16S rRNA gene, the entire internal transcribed spacer region, and a partial sequence of the 5.8S rRNA gene by using neighbor-joining analyses and computed relationships between sequences by using the Tamura 3-parameter method, gamma distribution, and parametric bootstrap analysis of 1,000 replicates in MEGA X software. 

### Microscopic Examination

We examined microsporidia PCR-positive samples microscopically. We prepared slides by mechanically homogenizing tissue samples with a mortar and pestle and centrifuged aspirates at 13,000 × *g* for 10 min; we stained aspirate sediments and homogenized tissues with Calcofluor M2R (Sigma Aldrich, https://www.sigmaaldrich.com) (*17*).

### Ethics Statement

We analyzed existing specimens beyond routine microbiologic screening, focusing on verifying the association between inflammatory disease and the presence of microsporidia in inflammatory foci. Because the study was performed by using samples with no human intervention arm, patient consent was not required.

## Results

We screened a total of 94 samples from 49 patients who were 41–96 (median 71) years of age for microsporidia infection in tissues surrounding the operated hip and knee joints. The mean age was 71 (SD+9.3; range 41–84) among hip replacement patients and 70 (SD+9.0; range 62–96) years among knee replacement patients. The male to female ratio was 12 (36%) to 21 (64%) in the hip replacement group and 9 (56%) to 7 (44%) in knee replacement group. Most patients had prosthetic joint infections; only 3 patients had other diagnoses, and the remaining patients had aseptic implant loosening (Table 1). Laboratory examinations showed physiologic indicators were within reference ranges for all patients. Patients did not undergo immunosuppressive treatment during the study period.

**Table 1:** Sample types, age of patients, surgical procedures, and diagnoses in study of microsporidia (*Encephalitozoon cuniculi*) in patients with degenerative hip and knee disease, Czech Republic*

Sample type	Periprosthetic joint infection				Aseptic implant loosening				Other diagnosis		
	NP/NS	PR/SR/TR	Mean age (SD)		NP/NS	PR/SR/TR	Mean age (SD)		NP/NS	PR/SR/TR	Mean age (SD)
Knees											
Joint fluid	7/8	4/2/1	72.7 (5.7)		NA	NA	NA		NA	NA	NA
Puncture aspirate	9/12	5/2/2	76.3 (8.9)		1/1	0/1/0	74		1/1	1/0/0	78
Joint tissue	9/14	5/1/3	73.0 (6.9)		NA	NA	NA		NA	NA	NA
Hips											
Joint fluid	11/13	6/2/3	68.3 (9.4)		9/9	5/3/1	69.1 (8.5)		NA	NA	NA
Puncture aspirate	7/8	6/0/1	66.9 (12.7)		1/1	1/0/0	74		NA	NA	NA
Joint tissue	10/17	5/2/3	71.9 (6.1)		6/8	5/1/0	70.5 (7.7)		2/2	0/0/2	69.5 (0.5)

*Samples were collected from immunocompetent patients during May 2020–September 2021 at Bulovka Hospital in Prague, Czech Republic. NA, not applicable; NP, number of patients; NS, number of samples; PR, primary revision; SR, secondary revision; TR, third and further revision.

**Figure 1:**
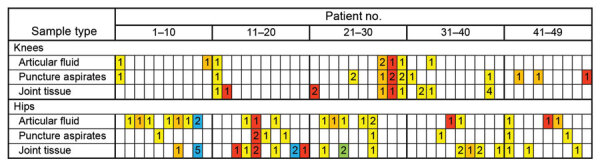
Samples obtained from each patient during revision surgery in study of microsporidia (*Encephalitozoon cuniculi*) in patients with degenerative hip and knee disease, Czech Republic. Samples were collected from immunocompetent patients during May 2020–September 2021 at Bulovka Hospital in Prague, Czech Republic. Numbers indicate the number of collected samples for each patient. Colors indicate the type of revision surgery: yellow, primary revision; orange, secondary revision; red, third and further revision; blue, both primary and third and further revision; green, both primary and secondary revision.

Among screened patients, 16 underwent knee arthroplasty providing 36 samples, and 33 underwent hip arthroplasty providing 58 samples (Table 1). The number of samples obtained from patients was 1–7; multiple samples mostly represented more sample types (Figure 1). Most (n = 28) patients underwent primary revision, then secondary and further revisions (9 each); 3 patients underwent repeated surgery: primary/secondary revision (patient no. 24) and primary/third and further revision (patient nos. 9 and 19) (Figure 1).

Of the 94 samples examined, most (61) were microbiologically sterile, whereas 12 samples were positive for *S. aureus* (5 were methicillin resistant), 6 were positive for *Escherichia coli*, 3 were positive for *E. faecalis*, 3 were positive for *Salmonella* Enteritidis, 2 were positive for *Staphylococcus epidermidis*, and 2 were positive for group G beta-hemolytic *Streptococcus*. *Streptococcus agalactiae*, *Corynebacterium tuberculostearicum*, *Pseudomonas aeruginosa*, or *Enterococcus faecium* were detected in the remaining samples.

**Figure 2:**
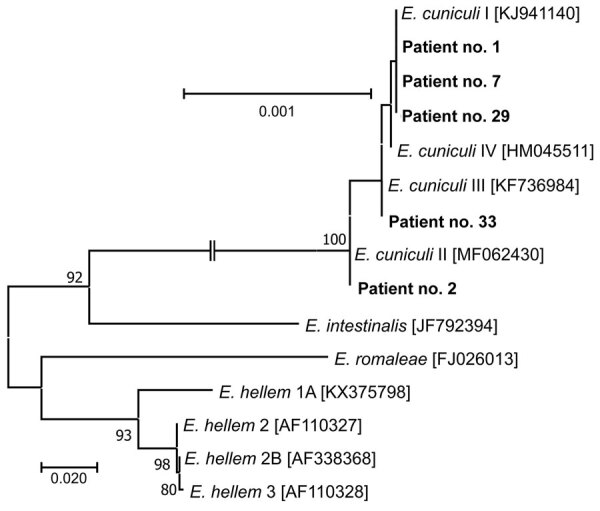
Phylogenetic analysis of *Encephalitozoon cuniculi* genotypes recovered from immunocompetent patients in study of microsporidia in patients with degenerative hip and knee disease, Czech Republic. Samples were collected from patients during May 2020–September 2021 at Bulovka Hospital in Prague. Partial sequences of 16S rRNA gene, the entire internal transcribed spacer region, and a partial sequence of 5.8S rRNA gene were inferred by using neighbor-joining analyses, and relationships were computed by using the Tamura 3-parameter method with gamma distribution and parametric bootstrap analysis of 1,000 replicates in MEGA X software (MEGA, https://www.megasoftware.net). Bold type indicates sequences obtained in this study, identified by patient number. Sequences for comparisons were obtained from GenBank; accession numbers are in brackets. Scale bar indicates nucleotide substitutions per site.

*Encephalitozoon*-specific DNA was confirmed in samples from 3 men and 2 women who were 63–78 years of age. Phylogenetic analyses revealed *E. cuniculi* genotypes I, II, and III. The 5 sequences obtained in this study were 100% identical to GenBank sequences for *E. cuniculi* genotype I (accession no. KJ941140), II (accession no. MF062430), and III (accession no. KF736984) (Figure 2).

**Figure 3:**
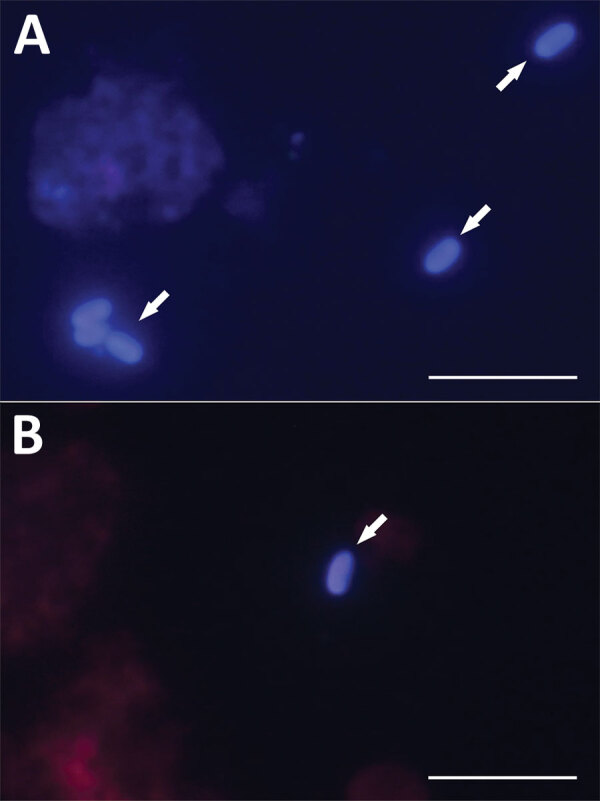
Microscopic analysis of *Encephalitozoon cuniculi* spores isolated from immunocompetent patients in study of microsporidia in patients with degenerative hip and knee disease, Czech Republic. Samples were collected from patients who tested positive for *Encephalitozoon* DNA during May 2020–September 2021 at Bulovka Hospital in Prague. Visualization of *E. cuniculi* from sample of knee joint fluid from patient no. 29 (A) and hip joint fluid from patient no. 2 (B). Arrows indicate *E. cuniculi* spores stained with Calcofluor M2R (Sigma Aldrich, https://www.sigmaaldrich.com) and viewed after fluorescence excitation at 490 nm wavelength. Scale bars are 10 μm.

We detected microsporidia in knee or hip aspirates obtained during ambulatory puncture and joint fluids and tissues recovered intraoperatively for all 5 *Encephalitozoon*-positive patients (Table 2). Of those 5 patients, 3 had periprosthetic joint infection, and 2 had aseptic implant loosening. *E. cuniculi* genotype I was most often detected, in 8 knee and hip samples from 3 patients; the number of spores ranged from 12 to 5,600 per gram of sample. We detected *Encephalitozoon cuniculi* genotype II in a hip sample (260 spores/g sample) from 1 patient, and genotype III in a knee sample (6.9 spores/g sample) from 1 other patient (Table 2). Microscopic analysis of Calcofluor M2R–stained smears confirmed the presence of spores (2–5 spores per slide) in tissue samples obtained from patient nos. 2 and 29 who tested positive for *Encephalitozoon* DNA (Figure 3). Samples from the other 3 patients were microscopically negative for spores. Microbiologic tests showed bacterial infections within the tissues of 3 patients: group G beta-hemolytic *Streptococcus* in the knee of patient no. 1, *E. faecalis* in the knee of patient no. 29, and methicillin-resistant *S. aureus* in the hip of patient no. 2; the other 2 patients were clinically classified as aseptic (Table 2).

**Table 2:** Characteristics of immunocompetent patients and patient samples in study of microsporidia (*Encephalitozoon cuniculi*) in patients with degenerative hip and knee disease, Czech Republic*

Patient no.	Age, y/sex	Samples					
		Origin	Pathology	Total/no. positive†	Genotype‡	No. spores/g sample (Ct)§	Microbiology
1	78/M	Knee, PR, puncture aspirate, fluid	PJI	2/2	I	74 (37)	Group G beta-hemolytic *Streptococcus*
29	76/M	Knee, TR, 2 puncture aspirates, fluid, tissue	PJI	4/4	I	5,600 (33)	*Enterococcus faecalis*
33	63/F	Knee, PR, fluid, tissue	AIL	2/2	III	6.9 (39)	Aseptic
2	75/M	Hip, PR, fluid	PJI	1/1	II	260 (35)	*Staphylococcus aureus* (MRSA)
7	71/F	Hip, PR, fluid, tissue	AIL	2/2	I	12 (38)	Aseptic

*Samples were collected during May 2020–September 2021 at Bulovka Hospital in Prague, Czech Republic. AIL, aseptic implant loosening; Ct, cycle threshold; MRSA, methicillin-resistant *Staphylococcus aureus*; PJI, periprosthetic joint infection; PR, primary revision; TR, third and further revision. †Total number of samples/number of *E*. *cuniculi*-positive samples. ‡*E*. *cuniculi* genotype was determined by nested PCR of partial sequence of the 16S rRNA gene that included the entire internal transcribed spacer. §Number of spores was determined by quantitative real-time PCR of a 268-bp region of the *E. cuniculi* 16S rRNA gene. DNA was isolated from tissue or aspirate sediment.

## Discussion

Primary hip and knee arthroplasty ranks among the top 5 most common procedures performed and among the top 5 fastest growing procedures each year across all surgical disciplines (*20*). Total joint replacement improves function, reduces pain, and improves quality of life for patients, and is cost-effective (*21*,*22*). Despite the high success rate of modern total joint arthroplasty (*23*) and technologic advances designed to extend the lifetime of primary implants (*24*–*26*), modern implant bearings and well-fixed components have a finite lifespan (*27*). Total joint replacements because of osteoarthritis require a revision procedure in 10% of patients, ≈4% within 10 years of initial surgery (*13*,*14*). Risk for revision increases in younger, more active patients and in those who have a higher body mass index (*28*). The most common reasons for revision surgery are infection, fracture around the implant, and loosening of the implant, which can occur soon after joint replacement or after decades of good function (*29*).

Prosthetic joint infection was detected in 34 (69.3%) of 49 patients we screened. Gram-positive cocci, such as *S. aureus*, coagulase-negative staphylococci, and *E. faecalis* are the major prosthetic joint infection-related microorganisms, after which Gram-negative bacilli are common (*30*–*32*); however, other pathogens are often overlooked, leading to an aseptic joint diagnosis. Microsporidia are often overlooked, fungus-related, obligate intracellular parasites occurring worldwide and infecting various vertebrate and invertebrate hosts, including humans (*33*,*34*); 17 species have been reported in humans, causing more severe symptoms in immunocompromised persons than in immunocompetent counterparts (*35*,*36*). *E. cuniculi* was the first microsporidium identified in mammals and the best-studied, forming the foundation of knowledge about microsporidia. *E. cuniculi* is typically described as a chronic, slow-acting pathogen and, thus, is considered less virulent than other pathogen groups; however, it can multiply successfully and extensively without any obvious signs of infection in immunocompetent hosts (*37*–*39*). *E*. *cuniculi* infects a wide spectrum of host cells, including epithelial cells, vascular endothelial cells, kidney tubule cells, and can be found in most tissues, having a propensity toward brain and kidneys (*40*). *E*. *cuniculi* is responsible for various pathologies depending on the infection site, affecting the nervous system as well as the respiratory and digestive tracts and causing hepatitis, peritonitis, pneumonitis, cystitis, nephritis, and encephalitis (*41*,*42*). Most documented cases originated from HIV/AIDS patients and transplant recipients. Whereas infection with *E. cuniculi* genotype I and II is common, occurrence of genotypes III and IV in humans is rare (*43*). As researchers and clinicians become more aware of those pathogens and are able to diagnose infections caused by them, new associations between microsporidia parasites and common infections have been reported (*17*,*44*). Moreover, *E. cuniculi* is able to survive and replicate in a variety of immune cells, including resident and migratory macrophages and other phagocytic cells, such as neutrophils, eosinophils, monocytes, and dendritic cells; thus, those immune cells might contribute to dissemination of *E. cuniculi* throughout the host organism (*45*,*46*).

The most common route of microsporidia transmission is the fecal-oral route; spores are passed in the urine or feces of infected persons into the environment and transmitted mostly through contaminated water sources (*43*). Microsporidia spores have been identified in wastewater, and in surface, irrigation, and drinking water. Moreover, several studies have reported foodborne transmission through fresh produce, such as strawberries, raspberries, lettuce, celery, parsley, and oranges, including orange juice. Recently, *E. cuniculi* has been reported in milk from dairy cows and goats, and the possibility of *E. cuniculi* transmission through pasteurized cow’s milk, fermented pork products, and fresh goat cheese has been experimentally documented (*43*). Furthermore, infection in the respiratory tract suggests airborne transmission by contaminated aerosols (*43*).

*E*. *cuniculi* can survive and persist in immunocompetent hosts, even after chemotherapeutic treatment (*47*–*49*), and a latent infection can be activated by inflammation in the host body (*9*). A role for proinflammatory immune cells in the expansion of *E. cuniculi* infection in host tissues has been suggested because of the occurrence of microsporidia in inflamed tissues (*17*) and the targeted migration toward inflammatory foci seen after experimental induction of inflammation (*9*,*10*). Thus, the incidence of microsporidia infections might be much higher than previously reported, and microsporidia might represent a neglected etiologic agent for more common diseases, including prosthetic joint infection. We confirmed periprosthetic *E. cuniculi* infection in 3 patients who had prosthetic joint infection and 2 who had aseptic implant loosening. Moreover, the molecular data were supported by microscopy in 2 patients who had the highest spore loads. The other 3 *E. cuniculi* PCR-positive patients had negative microscopic results; those results were likely caused by limited sensitivity of microscopy in samples with low spore load rather than laboratory contamination of PCR. Because we obtained uniform results from multiple samples from specific patients by using both PCR and quantitative PCR, it is unlikely that contamination occurred in all samples from a particular patient at the same time and not in other samples. Laboratory contamination was excluded as a possible reason for our results because the samples were taken and PCR was performed under sterile conditions by the same trained personnel, and the PCR diagnostics workspace is structurally divided into separate areas adhering to a one-direction workflow.

Whether microsporidia infection occurred in the affected joint areas before the onset of inflammatory processes or whether they entered the affected areas secondarily through macrophages or other cells involved in inflammation remains unclear. Nevertheless, not only infective agents can induce inflammation. Implant-derived wear particles can also induce host inflammatory responses via opsonization by danger-associated molecular pattern molecules and recognition by Toll-like receptors (*50*). Therefore, *E. cuniculi* spores likely were transported to the joints within immune cells associated with proinflammatory immune responses.

In conclusion, *E. cuniculi* can occupy unusual extraintestinal locations, such as joint fluid or tissue, and should be considered a contributing cause of joint inflammation and arthrosis. However, the role of this pathogen in causing osteolysis and subsequent implant loosening needs to be clarified. The presence of microsporidia spores and DNA in periprosthetic tissue of immunocompetent hosts indicates active infection in those patients and should be considered in the history of the disease. In addition, microsporidia should be considered as a potential cause of periprosthetic osteolysis and implant destabilization after hip replacement.

## References

[1] Cali A, Becnel JJ, Takvorian PM. Microsporidia. In: Archibald JM, Simpson AGB, Slamovits CH, editors. Handbook of the protists, 2nd edition. Cham (CH): Springer; 2017. p. 1559–618.

[2] Edlind TD, Li J, Visvesvara GS, Vodkin MH, McLaughlin GL, Katiyar SK. Phylogenetic analysis of beta-tubulin sequences from amitochondrial protozoa. Mol Phylogenet Evol. 1996;5:359–67. 10.1006/mpev.1996.0031 8728394

[3] Keeling PJ, Doolittle WF. Alpha-tubulin from early-diverging eukaryotic lineages and the evolution of the tubulin family. Mol Biol Evol. 1996;13:1297–305. 10.1093/oxfordjournals.molbev.a025576 8952074

[4] Wittner M. Historic perspective on the microsporidia: expanding horizons. In: Wittner M, Weiss LM, editors. The microsporidia and microsporidiosis. Washington DC: American Association of Microbiology; 1999. p. 1–6.

[5] Didier ES, Didier PJ, Snowden KF, Shadduck JA. Microsporidiosis in mammals. Microbes Infect. 2000;2:709–20. 10.1016/S1286-4579(00)00354-3 10884622

[6] Sak B, Kučerová Z, Kváč M, Květoňová D, Rost M, Secor EW. Seropositivity for *Enterocytozoon bieneusi*, Czech Republic. Emerg Infect Dis. 2010;16:335–7. 10.3201/eid1602.090964 20113575 PMC6110333

[7] Didier ES. Microsporidiosis: an emerging and opportunistic infection in humans and animals. Acta Trop. 2005;94:61–76. 10.1016/j.actatropica.2005.01.010 15777637

[8] Didier ES, Weiss LM. Microsporidiosis: not just in AIDS patients. Curr Opin Infect Dis. 2011;24:490–5. 10.1097/QCO.0b013e32834aa152 21844802 PMC3416021

[9] Brdíčková K, Sak B, Holubová N, Květoňová D, Hlásková L, Kicia M, et al. *Encephalitozoon cuniculi* genotype II concentrates in inflammation foci. J Inflamm Res. 2020;13:583–93. 10.2147/JIR.S271628 33061524 PMC7524191

[10] Sak B, Holubová N, Květoňová D, Hlásková L, Tinavská J, Kicia M, et al. Comparison of the concentration of *Encephalitozoon cuniculi* genotypes I and III in inflammatory foci under experimental conditions. J Inflamm Res. 2022;15:2721–30. 10.2147/JIR.S363509 35502243 PMC9056047

[11] Lallo MA, da Costa LFV, de Castro JM. Effect of three drugs against *Encephalitozoon cuniculi* infection in immunosuppressed mice. Antimicrob Agents Chemother. 2013;57:3067–71. 10.1128/AAC.00157-13 23612191 PMC3697356

[12] Kurtz SM, Lau E, Ong K, Zhao K, Kelly M, Bozic KJ. Future young patient demand for primary and revision joint replacement: national projections from 2010 to 2030. Clin Orthop Relat Res. 2009;467:2606–12. 10.1007/s11999-009-0834-6 19360453 PMC2745453

[13] Sloan M, Premkumar A, Sheth NP. Projected volume of primary total joint arthroplasty in the U.S., 2014 to 2030. J Bone Joint Surg Am. 2018;100:1455–60. 10.2106/JBJS.17.01617 30180053

[14] Malchau H, Garellick G, Berry D, Harris WH, Robertson O, Kärrlholm J, et al. Arthroplasty implant registries over the past five decades: Development, current, and future impact. J Orthop Res. 2018;36:2319–30. 10.1002/jor.24014 29663575

[15] Fernandez-Sampedro M, Salas-Venero C, Fariñas-Álvarez C, Sumillera M, Pérez-Carro L, Fakkas-Fernandez M, et al. 26Postoperative diagnosis and outcome in patients with revision arthroplasty for aseptic loosening. BMC Infect Dis. 2015;15:232. 10.1186/s12879-015-0976-y 26084830 PMC4470055

[16] Hodges NA, Sussman EM, Stegemann JP. Aseptic and septic prosthetic joint loosening: Impact of biomaterial wear on immune cell function, inflammation, and infection. Biomaterials. 2021;278:121127. 10.1016/j.biomaterials.2021.121127 34564034

[17] Kicia M, Wesolowska M, Kopacz Z, Kvác M, Sak B, Sokulska M, et al. Disseminated infection of *Encephalitozoon cuniculi* associated with osteolysis of hip periprosthetic tissue. Clin Infect Dis. 2018;67:1228–34. 10.1093/cid/ciy256 29659738

[18] Osmon DR, Berbari EF, Berendt AR, Lew D, Zimmerli W, Steckelberg JM, et al.; Infectious Diseases Society of America. Diagnosis and management of prosthetic joint infection: clinical practice guidelines by the Infectious Diseases Society of America. Clin Infect Dis. 2013;56:e1–25. 10.1093/cid/cis803 23223583

[19] Fillingham YA, Della Valle CJ, Suleiman LI, Springer BD, Gehrke T, Bini SA, et al. Definition of successful infection management and guidelines for reporting of outcomes after surgical treatment of periprosthetic joint infection: from the workgroup of the Musculoskeletal Infection Society (MSIS). J Bone Joint Surg Am. 2019;101:e69. 10.2106/JBJS.19.00062 31318814

[20] Fingar KR, Stocks C, Weiss AJ, Steiner CA. Statistical brief #186. Most frequent operating room procedures performed in U.S. Hospitals, 2003–2012. In Healthcare Cost and Utilization Project (HCUP) statistical briefs. Rockville (MD): Agency for Healthcare Research and Quality; 2006. 25695123

[21] Price AJ, Longino D, Rees J, Rout R, Pandit H, Javaid K, et al. Are pain and function better measures of outcome than revision rates after TKR in the younger patient? Knee. 2010;17:196–9. 10.1016/j.knee.2009.09.003 20133136

[22] Dakin H, Gray A, Fitzpatrick R, Maclennan G, Murray D; KAT Trial Group. Rationing of total knee replacement: a cost-effectiveness analysis on a large trial data set. BMJ Open. 2012;2:e000332. 10.1136/bmjopen-2011-000332 22290396 PMC3269047

[23] Quintana JM, Arostegui I, Escobar A, Azkarate J, Goenaga JI, Lafuente I. Prevalence of knee and hip osteoarthritis and the appropriateness of joint replacement in an older population. Arch Intern Med. 2008;168:1576–84. 10.1001/archinte.168.14.1576 18663171

[24] Lim SJ, Jang SP, Kim DW, Moon YW, Park YS. Primary ceramic-on-ceramic total hip arthroplasty using a 32-mm ceramic head with a titanium-alloy sleeve. Clin Orthop Relat Res. 2015;473:3781–7. 10.1007/s11999-015-4374-y 26024582 PMC4626516

[25] Delaunay CP, Putman S, Puliéro B, Bégin M, Migaud H, Bonnomet F. Cementless total hip arthroplasty with Metasul bearings provides good results in active young patients: a concise followup. Clin Orthop Relat Res. 2016;474:2126–33. 10.1007/s11999-016-4920-2 27278679 PMC5014817

[26] Sobieraj M, Marwin S. Ultra-high-molecular-weight polyethylene (UHMWPE) in total joint arthroplasty. Bull Hosp Jt Dis (2013). 2018;76:38–46. 29537956

[27] Schwartz AM, Farley KX, Guild GN, Bradbury TL Jr. Projections and epidemiology of revision hip and knee arthroplasty in the United States to 2030. J Arthroplasty. 2020;35(6S):S79–85. 10.1016/j.arth.2020.02.030 32151524 PMC7239745

[28] Bayliss LE, Culliford D, Monk AP, Glyn-Jones S, Prieto-Alhambra D, Judge A, et al. The effect of patient age at intervention on risk of implant revision after total replacement of the hip or knee: a population-based cohort study. Lancet. 2017;389:1424–30. 10.1016/S0140-6736(17)30059-4 28209371 PMC5522532

[29] Rabiu AR, Rasidovic D, Parsons H, Wall PDH, Metcalfe A, Bruce J. Surgical interventions for failed primary knee replacement. Cochrane Database Syst Rev. 2020;2020:CD013681.

[30] Zimmerli W, Trampuz A, Ochsner PE. Prosthetic-joint infections. N Engl J Med. 2004;351:1645–54. 10.1056/NEJMra040181 15483283

[31] Martínez-Pastor JC, Muñoz-Mahamud E, Vilchez F, García-Ramiro S, Bori G, Sierra J, et al. Outcome of acute prosthetic joint infections due to gram-negative bacilli treated with open debridement and retention of the prosthesis. Antimicrob Agents Chemother. 2009;53:4772–7. 10.1128/AAC.00188-09 19687237 PMC2772308

[32] Hsieh PH, Lee MS, Hsu KY, Chang YH, Shih HN, Ueng SW. Gram-negative prosthetic joint infections: risk factors and outcome of treatment. Clin Infect Dis. 2009;49:1036–43. 10.1086/605593 19691430

[33] Snowden KF. Microsporidia in higher vertebrates. In: Weiss LM, Becnel JJ, editors. Microsporidia: pathogens of opportunity, 1st edition. Chichester (UK): John Wiley & Sons, Inc.; 2014. p. 469–91.

[34] Fayer R, Santin‐Duran M. Epidemiology of microsporidia in human Infections. In: Weiss LM, Becnel JJ, editors. Microsporidia: pathogens of opportunity. Chichester (UK): John Wiley & Sons, Inc.; 2014. p. 135–64.

[35] Vávra J, Lukeš J. Microsporidia and ‘the art of living together’. Adv Parasitol. 2013;82:253–319. 10.1016/B978-0-12-407706-5.00004-6 23548087

[36] Didier ES, Khan IA. The immunology of microsporidiosis in mammals. In: Weiss LM, Becnel JJ, editors. Microsporidia: pathogens of opportunity. Chichester (UK): John Wiley & Sons, Inc.; 2014. p. 307–26.

[37] Sak B, Kotková M, Hlásková L, Kváč M. Limited effect of adaptive immune response to control encephalitozoonosis. Parasite Immunol. 2017;39:e12496. 10.1111/pim.12496 29032596

[38] Sak B, Brdíčková K, Holubová N, Květoňová D, Hlásková L, Kváč M. *Encephalitozoon cuniculi* genotype III evinces a resistance to albendazole treatment in both immunodeficient and immunocompetent mice. Antimicrob Agents Chemother. 2020;64:e00058–20. 10.1128/AAC.00058-20 32152088 PMC7179643

[39] Kotková M, Sak B, Kváč M. Differences in the intensity of infection caused by *Encephalitozoon cuniculi* genotype II and III - Comparison using quantitative real-time PCR. Exp Parasitol. 2018;192:93–7. 10.1016/j.exppara.2018.07.019 30075234

[40] Gannon J. A survey of *Encephalitozoon cuniculi* in laboratory animal colonies in the United Kingdom. Lab Anim. 1980;14:91–4. 10.1258/002367780780942917 6776345

[41] Mertens RB, Didier ES, Fishbein MC, Bertucci DC, Rogers LB, Orenstein JM. *Encephalitozoon cuniculi* microsporidiosis: infection of the brain, heart, kidneys, trachea, adrenal glands, and urinary bladder in a patient with AIDS. Mod Pathol. 1997;10:68–77. 9021729

[42] Weber R, Bryan RT, Schwartz DA, Owen RL. Human microsporidial infections. Clin Microbiol Rev. 1994;7:426–61. 10.1128/CMR.7.4.426 7834600 PMC358336

[43] Sak B, Kváč M. Chronic infections in mammals due to microsporidia. Exp Suppl. 2022;114:319–71. 10.1007/978-3-030-93306-7_12 35544008

[44] Ditrich O, Chrdle A, Sak B, Chmelík V, Kubále J, Dyková I, et al. *Encephalitozoon cuniculi* genotype I as a causative agent of brain abscess in an immunocompetent patient. J Clin Microbiol. 2011;49:2769–71. 10.1128/JCM.00620-11 21593268 PMC3147860

[45] Couzinet S, Cejas E, Schittny J, Deplazes P, Weber R, Zimmerli S. Phagocytic uptake of *Encephalitozoon cuniculi* by nonprofessional phagocytes. Infect Immun. 2000;68:6939–45. 10.1128/IAI.68.12.6939-6945.2000 11083817 PMC97802

[46] Nassonova ES, Tokarev YS, Trammer T, Entzeroth R, Sokolova YY. Phagocytosis of *Nosema grylli* (Microsporida, Nosematidae) spores in vivo and in vitro. J Eukaryot Microbiol. 2001;48(Suppl):83S–4S. 10.1111/j.1550-7408.2001.tb00462.x 11906090

[47] Sak B, Brady D, Pelikánová M, Květoňová D, Rost M, Kostka M, et al. Unapparent microsporidial infection among immunocompetent humans in the Czech Republic. J Clin Microbiol. 2011;49:1064–70. 10.1128/JCM.01147-10 21191056 PMC3067711

[48] Sak B, Kváč M, Kučerová Z, Květoňová D, Saková K. Latent microsporidial infection in immunocompetent individuals - a longitudinal study. PLoS Negl Trop Dis. 2011;5:e1162. 10.1371/journal.pntd.0001162 21629721 PMC3101169

[49] Kotková M, Sak B, Květoňová D, Kváč M. Latent microsporidiosis caused by *Encephalitozoon cuniculi* in immunocompetent hosts: a murine model demonstrating the ineffectiveness of the immune system and treatment with albendazole. PLoS One. 2013;8:e60941. 10.1371/journal.pone.0060941 23593356 PMC3623998

[50] Konttinen YT, Pajarinen J, Takakubo Y, Gallo J, Nich C, Takagi M, et al. Macrophage polarization and activation in response to implant debris: influence by “particle disease” and “ion disease”. J Long Term Eff Med Implants. 2014;24:267–81. 10.1615/JLongTermEffMedImplants.2014011355 25747030 PMC4373605

